# Differential Susceptibility to Antimony in Strains and Clinical Isolates of *Leishmania amazonensis* from Brazil: In Vitro and In Vivo Studies and Implications for Drug Response and Treatment Failure

**DOI:** 10.3390/pathogens15020220

**Published:** 2026-02-15

**Authors:** Victor de Sousa Agostino, Leonardo F. Geres, Stéphane de la Roca, Beatriz T. de Moraes, Juliana I. Aoki, Elizabeth M. Coser, Nilson Branco, Adriano C. Coelho

**Affiliations:** Departamento de Biologia Animal, Instituto de Biologia, Universidade Estadual de Campinas (UNICAMP), Campinas 13083-862, Brazil; victor.agostino@gmail.com (V.d.S.A.); leonardo.geres@outlook.com (L.F.G.);

**Keywords:** *Leishmania amazonensis*, cutaneous leishmaniasis, pentavalent antimony, treatment failure

## Abstract

In Brazil, cutaneous leishmaniasis is endemic and may be caused by *Leishmania amazonensis*. This species is the second most prevalent species in that country, and it is responsible for localized cutaneous and diffuse cutaneous leishmaniasis. Pentavalent antimony is still the first-line drug for cutaneous leishmaniasis treatment in Brazil. In this study, we investigated the in vitro susceptibility to antimony of a panel of *L. amazonensis* strains and clinical isolates responsible for cutaneous and diffuse cutaneous leishmaniasis. There was a significant variation in susceptibility to antimony not only within these strains and isolates evaluated at either promastigote or intracellular amastigote stages, but also between the two parasite stages for some of these strains and isolates. Additionally, we investigated whether this in vitro susceptibility variation to antimony would affect the in vivo response to treatment, using an experimental BALB/c mouse model of cutaneous leishmaniasis infected with three strains differing in susceptibility. Despite antimony could mildly reduce the lesion size in mice infected with one of these strains, no significant reduction in the parasite burden was found in treated animals, and they were completely refractory to drug treatment. These findings indicate that antimony treatment, even at high dosages via the intraperitoneal route, was not effective against *L. amazonensis* infection in this animal model. Finally, this study provides a preclinical dataset of the activity of antimony against a panel of strains and isolates of a species responsible for localized cutaneous and diffuse cutaneous leishmaniasis in Brazil.

## 1. Introduction

The parasitic protozoa of the genus *Leishmania* are the etiological agents of leishmaniasis, a spectrum of vector-borne diseases transmitted through bites from infected female phlebotomine sandflies. The disease has two main clinical manifestations: visceral and cutaneous leishmaniasis (CL). The latter may be classified as localized cutaneous leishmaniasis, the most common clinical form of CL, which eventually progresses to more serious manifestations such as mucocutaneous leishmaniasis, disseminated cutaneous leishmaniasis, and diffuse cutaneous leishmaniasis (DCL) in approximately 10% of patients [1]. The most endemic country in Latin America is Brazil, and seven species are responsible for clinical forms of CL: *Leishmania (Viannia) braziliensis,* which is the most prevalent species, as well as *L.* (*Leishmania*) *amazonensis*, *L.* (*V.*) *guyanensis*, *L.* (*V.*) *naiffi*, *L.* (*V.*) *lainsoni*, *L.* (*V.*) *shawi*, and *L.* (*V.*) *lindenbergi* [1,2]. In Brazil, 182,072 cases of CL were reported in the last ten years, corresponding to a prevalence of 89.6 cases per 100,000 inhabitants [3]. According to the most recent report of the Pan American Health Organization, 12,910 new cases of CL occurred in 2023 within Brazilian territory [4]. Among these cases of CL, 73.2% occurred in males and 41.5% in people aged from 20 to 39 years, with almost 45% of the patients not having completed elementary school. Regarding the clinical form, CL is responsible for 94% of cases, and only 6% were classified as mucocutaneous leishmaniasis, mainly associated with a sylvatic transmission pattern [3].

*Leishmania amazonensis* is a highly relevant species in Brazil, being the second most prevalent species in the country [2]. This species is responsible for localized CL, a clinical form characterized by a single or small number of lesions at the site of an infected sand fly bite, and DCL, which is characterized by a specific failure of the protective T-cell response [1,2]. In general, DCL shows a weak response to the available drugs for the disease.

Chemotherapy of leishmaniasis is restricted to a limited number of drugs, including pentavalent antimonials (SbV) in their two formulations, sodium stibogluconate (Pentostam) and meglumine antimoniate (Glucantime); amphotericin B (AmB), which also has two formulations, deoxycholate and the less toxic formulation, liposomal AmB; and pentamidine. Miltefosine is the only oral drug available for CL treatment, with cure rates higher than 70% in Brazil [5,6]. On the other hand, SbV is still considered the first-line treatment for CL, despite its cure rates of 50% or lower, the parenteral route administration, and high toxicity [5,7,8]. Due to this low response to the treatment, the recommended dosage of meglumine antimoniate (Glucantime) has increased in the last decades. The current treatment in Brazil consists of 20 mg/kg body weight per day with a period of administration of 28–30 days. Finally, both AmB formulations have cure rates higher than 80%, and their use is associated with cases of therapeutic failure and/or patients coinfected with the human immunodeficiency virus (HIV) [7,9].

The mechanism of action of SbV is partially known and has several targets in the parasite. It is a prodrug that needs to be reduced to the trivalent form (SbIII) for anti-*Leishmania* activity. This reduction occurs within the macrophage, the host cell of the parasite, and in amastigotes [10]. At least two enzymes are considered responsible for the conversion process: antimonite reductase and thiol-dependent reductase 1. A decrease in or loss of SbV to SbIII conversion, as well as an increase in trypanothione levels, the main thiol in the parasite that promotes parasite protection by increasing thiol redox potential, have already reported as mechanisms of drug resistance in *Leishmania* [10,11]. For example, the overexpression of enzymes such as ornithine decarboxylase and gamma-glutamylcysteine synthase, responsible for trypanothione biosynthesis, led to antimony resistance in *Leishmania* [12,13]. The SbIII affects energy metabolism due to the inhibition of glycolysis and fatty acid β-oxidation, causing a decrease in intracellular ATP and inducing the generation of reactive oxygen species [14]. It also affects trypanothione reductase activity, an enzyme involved in the parasite’s redox balance, leading to an increase in oxidative stress [15]. The active form of the drug enters the parasite via the Aquaglyceroporin 1 (AQP1), a transmembrane channel protein that transports water, non-polar solutes—such as urea or glycerol—and toxic metalloids such as SbIII [16]. Reduced levels of *AQP1* mRNA and/or mutations in the *AQP1* gene are associated with antimony resistance in resistant lines selected in vitro and clinical isolates that are resistant to antimonials [17,18,19]. Additionally, SbV may enter the parasite via an uncharacterized transporter that is then reduced to SbIII by amastigotes once inside the cell. ATP-binding cassette (ABC) transporters have been involved in antimony resistance. Within this family, MRPA is the main ABC transporter involved in antimony resistance by gene overexpression. It is localized in membrane vesicles close to the flagellar pocket and sequesters thiol-SbIII conjugates in intracellular vesicles [10,20].

A limited number of studies have investigated the correlation between clinical response and in vitro susceptibility to antimony [21,22,23,24,25,26,27]. For that reason, it is still unclear whether differential susceptibility to antimonials in vitro may be correlated with the in vivo response, using animal models, for example. Considering that SbV is widely used for the treatment of CL in Brazil, the main goal of this study was to determine the in vitro susceptibility of strains and clinical isolates of *L. amazonensis* from Brazil. To investigate whether the variable in vitro susceptibility to antimonials correlates with in vivo response, we also evaluated SbV effectiveness in treating mice infected with the least or most susceptible isolates, along with the reference strain of this species.

## 2. Materials and Methods

### 2.1. Leishmania amazonensis Strains and Clinical Isolates

In this study, the common strains of *L. amazonensis* were used: MHOM/BR/1973/M2269, MHOM/BR/1975/JOSEFA, IFLA/BR/1967/PH8, and MPRO/BR/1972/M1841 (also described as LV79). Five clinical isolates of *L. amazonensis* obtained from patients with CL at the Instituto de Infectologia Emílio Ribas, São Paulo, Brazil, were also used. These clinical isolates were previously typed by sequencing of the internal transcribed ribosomal spacer (ITS) DNA [28]. Additionally, the clinical isolate AAB (MHOM/BR/2019/AAB-MA) was isolated from a DCL patient coinfected with HIV and typed as *L. amazonensis* [29].

### 2.2. Culture Condition and Selection of a Sb-Resistant Line

Promastigotes of *L. amazonensis* strains and clinical isolates were maintained at 25 °C in 199 medium (M199) (Thermo Fisher Scientific, Waltham, MA, USA) supplemented with 40 mM HEPES (pH 7.4), 0.1 mM adenine, 5 μg/mL hemin, 10% heat-inactivated fetal bovine serum, 100 U/mL penicillin, and 100 μg/mL streptomycin [30].

A Sb-resistant line was obtained by exposing promastigotes of the M2269 strain to increasing SbIII concentrations, starting at 50 μM, until achieving a final concentration of 500 μM. Clonal lines were obtained from the SbIII-resistant population (Sb500) after plating onto M199 containing 1% agar (Thermo Fisher Scientific, Waltham, MA, USA). After 10–15 days, colonies were picked and expanded in liquid M199 containing SbIII.

### 2.3. Susceptibility Assays Against Promastigotes and Intracellular Amastigotes

In vitro susceptibility assays were performed using stock solutions of potassium antimony tartrate trihydrate (SbIII) (Sigma-Aldrich, Waltham, USA; 100 mM), and SbV (Glucantime, Sanofi-Aventis, Paris, France; 100 mM), both diluted in Milli-Q ultrapure water and then filter-sterilized (0.22 μm pore size).

The activity of SbIII against promastigotes of *L. amazonensis* strains, clinical isolates, and Sb-resistant clonal lines was evaluated using the MTT (3-(4,5-dimethylthiazol-2-yl)-2,5-diphenyltetrazoline bromide) colorimetric assay, as previously described [31]. Late log-phase promastigotes (2 × 10^6^ parasites per well) were incubated in the presence of SbIII serially diluted (1:2) for 24 h. The concentration range used (250 to 3.9 μM and/or 2000 to 31.25 μM) was adjusted based on the susceptibility of each strain or clinical isolate, varying accordingly. At least three independent experiments were performed in triplicate, and the 50% effective concentration (EC_50_) was determined by sigmoidal regression curves generated by the GraphPad Prism 8 software.

For intracellular amastigotes, the activity of SbV was determined as previously described [31]. Briefly, bone marrow-derived macrophages (BMDMs) were obtained from BALB/c mice and incubated in a 5% CO_2_ atmosphere at 37 °C [32]. Macrophages were then infected with stationary-phase promastigotes of strains, clinical isolates, and Sb-resistant lines at a ratio of 5:1 (parasites:macrophage) and incubated at 34 °C in a 5% CO_2_ atmosphere. After 3–4 h, non-internalized parasites were removed by washing with warmed PBS. Next, infected macrophages were treated with SbV serially diluted (1:2) (2000 to 50 μM) for 144 h, with an intermediate replacement of medium and SbV after 72 h of infection. The percentage of infection and number of amastigotes per infected macrophage were determined by counting at least 100 macrophages in three independent experiments that were used to determine EC_50_ values as described above.

### 2.4. BALB/c Mice Infection and Treatment with Sbv

For in vivo studies, female BALB/c mice (4–6 weeks) were used, obtained from the Centro Multidisciplinar para Investigação Biológica (CEMIB) of UNICAMP. Animals were kept in mini-isolators and received food and water ad libitum. For evaluation of SbV effectiveness, groups of five mice were randomly grouped and then infected with either the M2269 strain or the ER118 and AAB clinical isolates by inoculating 1 × 10^6^ stationary-phase promastigotes resuspended in 30 μL of filter-sterilized phosphate-buffered saline (PBS) into the right hind footpad. Treatment started at the 4th week post-infection, and the dosages used were 50 or 200 mg/kg/day of Glucantime (equivalent to 13.5 and 54 mg SbV/kg/day, respectively) via the intraperitoneal route for 15 days as previously described [33]. For all in vivo experiments, an untreated group infected with the M2269 strain or each clinical isolate was used as a control.

To evaluate SbV effectiveness, lesion sizes were measured weekly with a caliper (Mitutoyo Corporation, Kawasaki, Japan) and, at the end of the treatment, lesion tissues from each mouse were submitted to parasite burden quantification by quantitative real-time PCR, as described previously [33]. Histological analyses of lesions were conducted on isolated infected hind footpad fragments, which were fixed in formalin and then processed for paraffin embedding. Sections were stained with hematoxylin-eosin and then visualized under an optical microscope.

### 2.5. Statistical Analysis

All statistical analyses were performed using the GraphPad Prism 8 software. An unpaired two-tailed Student’s *t*-test was used to compare the EC_50_ values determined in vitro in the promastigote and intracellular amastigote stages for the strains and clinical isolates of *L. amazonensis*. Spearman’s correlation coefficient was calculated to determine the correlation between the EC_50_ values in the promastigotes and amastigotes of the strains and clinical isolates. The one-way ANOVA test and Tukey’s multiple comparison post-test were used to compare the parasite burden of groups of infected mice with either the M2269 strain or the ER118 and AAB isolates. The *p* value < 0.05 was considered statistically significant.

## 3. Results

### 3.1. In Vitro Susceptibility of L. amazonensis Strains and Clinical Isolates at Promastigote and Intracellular Amastigote Stages to Antimonials

**Figure 1:**
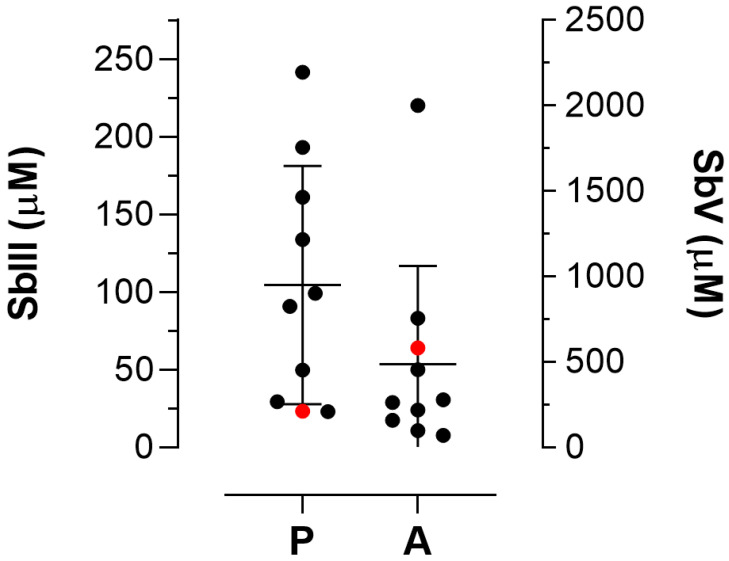
Susceptibility of promastigotes (P) and intracellular amastigotes (A) of strains and clinical isolates of *L. amazonensis* to antimonials. EC_50_ values of strains or isolates were determined in both stages of the parasite and are indicated in black circles. The EC_50_ values of the reference strain M2269 are indicated in red circles.

Initially, the activity of SbIII against the strains and clinical isolates of *L. amazonensis* in the promastigote stage was assessed. The EC_50_ values of strains and clinical isolates ranged from 23.4 ± 0.90 μM to 241.60 ± 4.81 μM (Table 1; Figure 1). Their infectivity was previously determined in BMDMs [34,35]; when reassessed, similar values were obtained (Table 1). The cytotoxicity of SbV against BMDMs, the host cell of intracellular amastigotes, was also previously determined; the CC_50_ value against BMDMs is >2000 μM after 72 h of treatment, rendering SbV non-toxic for these cells [35]. The EC_50_ values for the intracellular amastigotes of strains and isolates against SbV ranged from 71.69 ± 8.70 μM to >2000 μM (Table 1; Figure 1). In general, the strains and clinical isolates of *L. amazonensis* were more susceptible to SbIII at the promastigote stage when compared with intracellular amastigotes that were treated with SbV (Table 1; Figure 1). Despite using different drugs in the in vitro assays, no significant correlation between the EC_50_ values of the intracellular amastigote and promastigote stages was observed according to the Spearman correlation test (r = −0.1273, *p* = 0.733).

Considering the *L. amazonensis* M2269 strain as susceptible to antimony, the Activity Index (AI) values for SbV in strains and isolates ranged from 0.12 to 1.30. In fact, the only isolate whose AI was higher than 3 was the AAB isolate, whose EC_50_ at the intracellular amastigote stage was approximately 28-fold higher than the EC_50_ of the most susceptible isolate (ER118) (Table 1). This highly tolerant clinical isolate was obtained after the treatment of the patient with meglumine antimoniate (10 mg/kg/day), with no clinical cure [29]. On the other hand, the other isolates evaluated were obtained from patients before clinical treatment (Table 1). These patients were later treated by systemic administration of meglumine antimoniate (15 mg/kg/day for 20 days), with clinical improvement observed. Therefore, these isolates were not previously exposed to SbV, indicating that their susceptibility to antimonials is intrinsic.

To compare and evaluate the potential resistance phenotype to antimonials in strains and isolates of *L. amazonensis*, we generated Sb-resistant parasites through stepwise selection, using the M2269 strain at the promastigote stage as the parental line. The selection protocol began with an initial concentration of 50 μM SbIII, increasing up to 500 μM, after approximately 150 days in culture (Supplementary Figure S1). The Sb-resistant population, named Sb500, was cloned, and five independent clonal lines were obtained (Sb500.1, Sb500.2, Sb500.3, Sb500.4, and Sb500.5). In the promastigote stage, these clonal lines had a lower proliferation rate than the M2269 strain until the early stationary phase (Figure S2), and their EC_50_ values ranged from 549.16 to >2000 (Table 2).

Sb500.2 and Sb500.4 lines had lower infection rates in BMDMs than the parental line (M2269 strain) (Table 2). The average number of amastigotes per macrophage was 1.54 ± 0.25 and 1.72 ± 0.32 for the Sb500.2 and Sb500.4 lines, respectively, and 4.18 ± 0.41 for the M2269 strain. Both Sb-resistant lines were also resistant to SbV in the amastigote stage, with EC_50_ values at least 3.26-fold higher than those for the M2269 strain (Table 2). To evaluate whether the Sb-resistant phenotype persists in vivo, these two resistant lines were initially used to infect mice. However, no lesion in animals infected with these lines were observed after 4 weeks post-infection (Figure S3), leading us to disregard their use in in vivo studies.

### 3.2. In Vivo Treatment with SbV of Mice Infected with the L. amazonensis M2269 Strain and the ER118 and AAB Isolates

To investigate whether the in vitro Sb-resistance phenotype observed for the AAB isolate would translate in vivo, we investigated the efficacy of SbV via intraperitoneal administration in BALB/c mice infected with this isolate, as well as with the ER118 clinical isolate and the M2269 strain, which are highly and partially susceptible to antimonials, respectively (Table 1). It is important to state that despite the high dosages used for treatment, we did not observe any sign of potential toxicity in the treated mice, such as weight loss and cachexia, lethargy, or piloerection.

**Figure 2:**
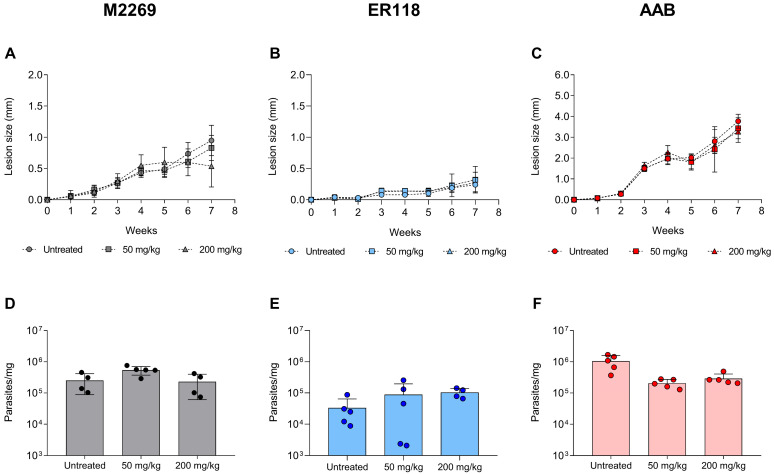
In vivo assessment of SbV activity against *L. amazonensis* infections in mice. Groups of five mice were infected with stationary-phase promastigotes of the *L. amazonensis* M2269 reference strain (**A**,**D**), the ER118 clinical isolate (**B**,**E**), or the AAB clinical isolate (**C**,**F**). Lesion size was monitored weekly throughout the course of infection (**A**–**C**). SbV (Glucantime) treatment was initiated on the 4th week post-infection with dosages of 50 or 200 mg/kg/day of SbV, administered intraperitoneally for 15 consecutive days. Parasite burden was determined at the end of the treatment period (7th week post-infection) by quantitative real-time PCR analysis of infected tissues (**D**–**F**). Data were analysed using one-way ANOVA followed by Tukey’s multiple comparison tests.

We found significant differences in the lesion size progression, particularly in animals infected with the AAB isolate, which had larger lesions than animals infected with either one of the other strains in the untreated groups (Figure 2A–C). Animals infected with the M2269 strain were refractory to SbV, with no reduction of parasite burden in the lesions of animals treated with 50 and 200 mg/kg/day (Figure 2A,D), despite a small lesion size reduction for the group treated with the higher dose of SbV. Similar findings were observed for animals infected with ER118 and AAB isolates, which were also refractory to both SbV dosages (Figure 2E,F). For these groups, the animals presented similar progression of the lesion and parasite burden at the end of the treatment as compared with the corresponding untreated groups (Figure 2B,C,E,F). Finally, histological analysis of tissues at the site of infection confirmed the presence of intracellular amastigotes in SbV-treated mice infected with the M2269 strain, as well as AAB and ER118 isolates (Figure S4), corroborating the results obtained for lesion size and parasite burden. For that reason, the differential susceptibility to antimonials found in vitro for the clinical isolates and the M2269 strain could not be correlated to the drug response in vivo in this animal model.

## 4. Discussion

In this study, we investigated the in vitro activity of antimonials in a panel of strains and clinical isolates of *L. amazonensis* from Brazil. The susceptibility of promastigotes and intracellular amastigotes to SbIII and SbV, respectively, was compared to the M2269 strain, a WHO reference strain. We found significant variation in susceptibility to SbV in vitro depending on the clinical isolates and strains evaluated, particularly at the amastigote stage, which is responsible for the disease in humans. Additionally, there was no correlation in drug susceptibility between the two parasite stages. This was unexpected at first, given our previous findings showing the in vitro activity of paromomycin, amphotericin B, and miltefosine against a panel of clinical isolates and strains of several *Leishmania* species, including *L. amazonensis* [28,35,36], and the possibility of such a correlation. Unlike other antileishmanial drugs, SbV is a prodrug that cannot be metabolized by promastigotes, being reduced to SbIII by macrophages and intracellular amastigotes [10]. For that reason, SbIII is used for the activity against promastigotes, while SbV is used for assays using intracellular amastigotes. Consequently, it is likely to assume that the lack of correlation in susceptibility between the two parasites stages may be due to the fundamental differences between the drugs used for these assays. Therefore, these findings indicate that the susceptibility to SbIII in *L. amazonensis* promastigotes is not predictive of a similar activity against intracellular amastigotes for SbV.

Previous studies have shown that the susceptibility in vitro to antimonials varies depending on the causative species [14,23], and a limited number of strains and isolates of *L. amazonensis* have been evaluated so far [23]. Here, a total of 10 strains and isolates of this species were evaluated for susceptibility at both stages of the parasite. All clinical isolates were obtained from patients before clinical treatment with SbV, indicating that their susceptibility to the drug is intrinsic [35]. In all cases, patients were treated with SbV and displayed complete regression of lesions, despite their significant variation in in vitro susceptibility to Sb. Moreover, these isolates were not previously exposed to SbV, indicating that their susceptibility to the drug is intrinsic. The only exception was a highly tolerant isolate, AAB, obtained after the patient was treated with meglumine antimoniate without clinical cure [29]. In this case, the patient’s treatment failure could be related to the isolate’s resistance phenotype. The EC_50_ of this isolate at the intracellular amastigote stage was approximately 3- and 28-fold higher than the EC_50_ of the M2269 strain and of the most susceptible isolate (ER118), respectively. In addition, we also selected Sb-resistant lines through stepwise selection, using the M2269 strain as parental line. These resistant lines were used as a reference for the resistance phenotype in vitro by comparing their susceptibility to SbV to the other strains and isolates used in this study. Interestingly, we found that AAB isolate was as tolerant to SbV as the selected Sb-resistant lines, rendering this isolate equally resistant to antimonials. Unfortunately, we could not assess if these selected resistant lines would be resistant to SbV treatment, due to an incapacity to induce lesions on the footpads of the infected mice. This was possibly associated with the fitness loss for these Sb-resistant lines, probably related to the selection of resistant parasites. In contrast, despite the AAB isolate being highly resistant to SbV, it is highly virulent in vivo, causing larger lesions when compared with those caused by the M2269 strain and ER118 isolate. Similarly, in *L. donovani*, Sb-resistant clinical isolates had a greater capacity to cause disease in mice when compared to a Sb-susceptible isolate [37]. These findings indicate that the molecular basis of acquired resistance in clinical isolates may be different from those found in Sb-resistant lines selected in vitro, as already described in *L. panamensis* [38], and that this may affect the parasite fitness, as observed in this study.

Considering the differential in vitro SbV activity in the M2269 strain and the AAB and ER118 isolates at the amastigote stage, the effectiveness of this drug in vivo was evaluated in a murine experimental model to investigate whether the in vitro SbV susceptibility would be correlated with the treatment outcome. The M2269 strain, together with the most tolerant and susceptible isolates—AAB and ER118, respectively—were chosen for the in vivo infection of BALB/c mice. Surprisingly, all three isolates were refractory to the treatment regardless of their significant variation in in vitro susceptibility to SbV (ranging from 71.96 to >2000 μM). The highest dosage used in this in vivo assay was 10-fold higher than the dosage used for human treatment with SbV. Additionally, this dosage is within the dose translation protocol recommended by the Food and Drug Administration, which takes into consideration the body surface area of humans and mice [39]. For that reason, the unresponsiveness of these three isolates evaluated with differential in vitro susceptibility to SbV was an unexpected finding. Previous studies have already shown that lower dosages were unable to reduce parasite burden in this same mouse model infected with *L. amazonensis* and treated intraperitoneally [40,41]. On the other hand, the daily intralesional treatment of an infection at the base of the tail at a dosage of 50 mg/kg for 14 days led to a 98% parasite burden reduction [42], while mice infected with *L. amazonensis* in the ear and treated via an intraperitoneal dose of 1300 mg/kg twice a week for 28 days led to an approximate 94% reduction in parasite burden [43]. Therefore, SbV was unable to treat CL in mice when administered intraperitoneally, and the inefficacy of SbV to even mildly control the infection in the footpad has not been discussed in depth. It is known that mouse infection affects antimony pharmacokinetics and that higher levels of the drug accumulate in the infected rather than in the uninfected footpad when administered intraperitoneally [44]. The drug is accumulated in the liver, followed by a slow clearance via two elimination routes: a renal and a biliary excretion [44]. In humans, the absorption of the drug is as effective when administered intralesionally as when administered intramuscularly [45]. However, the concentration of SbV in the lesion of CL patients has high variability (ranging from 7.46 to 70.68 μg/g) after 20 days of therapy with dosages of 10–20 mg SbV/kg/day [46]. It would be interesting to investigate in infected mice whether the dosage administered affects the concentration of the SbV in the infected footpad.

Previously, animals infected with the M2269 strain or the AAB isolate and treated with paromomycin, miltefosine, or a combination of both drugs presented a similar reduction in lesion size and parasite burden, despite the resistance phenotype to amphotericin B in vitro and in vivo for AAB isolate [34]. This confirms that this murine model is responsive to other drug treatments. The AAB clinical isolate, obtained from a patient that was refractory to the treatment with SbV [29], was also resistant to SbV at the amastigote stage. Unfortunately, the absence of responsiveness to SbV treatment in mice infected with moderate and highly susceptible isolates did not allow us to conclude whether the resistance phenotype of the AAB isolate persists in vivo.

In conclusion, Brazilian clinical isolates and strains of *L. amazonensis* display a wide range of in vitro susceptibility to antimonials, particularly those never exposed to the drug before. These intrinsic differences, however, do not seem to predict clinical outcome, as all patients achieved clinical cure after SbV treatment, with the exception of the patient infected with the AAB isolate. An in-depth molecular analysis of the intrinsic differences between susceptible and tolerant strains/isolates could help to understand the molecular basis of resistance to SbV treatment. In addition, our findings raised questions regarding the use of an experimental mouse model of CL, which was refractory to SbV treatment, despite the wide range in antimony susceptibility among the parasites. Finally, a deeper understanding of how SbV acts in this mouse model of *L. amazonensis* infection is required to further explore its antileishmanial activity in vivo.

## Figures and Tables

**Table 1:** In vitro susceptibility of promastigotes and intracellular amastigotes of strains and clinical isolates of *L. amazonensis* to antimonials (SbIII or SbV).

Strain/Isolate	Clinical Form ^a^	Treatment (SbV Dosage) ^b^	Clinical Cure ^c^	EC_50_ [SbIII] (μM) ^d^	EC_50_ [SbV] (μM) ^d^		Infection Rate ^f^
				Promastigote	Amastigote	AI ^e^	
MHOM/BR/1973/M2269 ^g^	LCL	-	-	23.40 ± 0.90	581.70 ± 7.93	-	93 ± 4.3
MHOM/BR/1975/JOSEFA	LCL	-	-	49.67 ± 2.17	157.63 ± 47.53	0.27	90 ± 13.7
MPRO/BR/1972/M1841	-	-	-	23.13 ± 1.33	262.25 ± 25.39	0.45	77 ± 1.4
IFLA/BR/1967/PH8	-	-	-	241.60 ± 4.81	277.80 ± 15.20	0.48	98 ± 1.1
MHOM/BR/2008/ER054	LCL	15 mg/kg/day	Yes	133.93 ± 5.57	97.26 ± 11.87	0.17	31 ± 7.2
MHOM/BR/2009/ER117	MCL	15 mg/kg/day	Yes	99.08 ± 6.89	455.97 ± 51.80	0.78	43 ± 0.6
MHOM/BR/2009/ER118	LCL	15 mg/kg/day	Yes	90.71 ± 9.80	71.69 ± 8.70	0.12	38 ± 0.1
MHOM/BR/2012/ER256	LCL	15 mg/kg/day	Yes	193.20 ± 16.63	219.80 ± 43.46	0.38	68 ± 1.4
MHOM/BR/2008/UB017	LCL	15 mg/kg/day	Yes	161.03 ± 7.80	756.03 ± 78.12	1.30	90 ± 0.5
MHOM/BR/2019/AAB-MA	DCL	10 mg/kg/day	No	29.39 ± 4.62	>2000	>3.44	90 ± 1.5

^a^ LCL, localized cutaneous leishmaniasis; MCL, mucocutaneous leishmaniasis; DCL, diffuse cutaneous leishmaniasis. ^b^ Treatment by systemic administration of meglumine antimoniate (Glucantime) after isolation of clinical isolates, except for the patient infected with the AAB isolate, which was isolated after treatment [29,35]. ^c^ Cure after meglumine antimoniate treatment [29,35]. ^d^ EC_50_ ± standard deviation in μM of three independent experiments. ^e^ Activity Index (ratio between the EC_50_ of the isolate and EC_50_ of the M2269 strain at amastigote stage). ^f^ Percentage of infected macrophages. ^g^ Line highlighted in gray corresponds to the WHO reference strain of *L. amazonensis* used in this study. (-) Data not provided, not available, or not specified.

**Table 2:** In vitro susceptibility of promastigotes and intracellular amastigotes of Sb-resistant clonal lines of *L. amazonensis* to antimonials (SbIII or SbV).

Strain/Resistant Clonal Lines	EC_50_ [SbIII] (μM) ^a^	EC_50_ [SbV] (μM) ^a^		Infection Rate ^c^
	Promastigote	Amastigote	AI ^b^	
*L. amazonensis* M2269 ^d^	23.40 ± 0.90	581.70 ± 7.93	-	93 ± 4.3
Sb500.1	>2000	-	-	-
Sb500.2	549.16 ± 16.60	>2000	>3.44	43 ± 8.6
Sb500.3	>2000	-	-	-
Sb500.4	>2000	1898 ± 57.50	3.26	46 ± 9.8
Sb500.5	>2000	-	-	-

^a^ EC_50_ ± standard deviation in μM of three independent experiments. ^b^ Activity Index (ratio between the EC_50_ of the resistant clonal line and the EC_50_ of the M2269 strain at the amastigote stage). ^c^ Percentage of infected macrophages. ^d^ Line highlighted in gray corresponds to the parental strain of *L. amazonensis* used for the generation of Sb-resistant lines. (-) Data not determined.

## Data Availability

Data is contained within the article or Supplementary Material.

## Supplementary Materials

The following supporting information can be downloaded at: https://www.mdpi.com/article/10.3390/pathogens15020220/s1. Figure S1: Schematic representation of in vitro selection of Sb-resistant promastigotes through stepwise selection; Figure S2: Promastigote proliferation of the M2269 strain, and Sb-resistant lines (Sb500.1 to Sb500.5); Figure S3: Progression of lesion size in infected mice with the M2269 strain and Sb-resistant clonal lines (Sb500.2 or Sb500.4) over the weeks; Figure S4: Histological analysis of the infected mice with *L. amazonensis* M2269 strain, or the ER118 and AAB clinical isolates.

## Abbreviations

The following abbreviations are used in this manuscript:

ABC	ATP-binding cassette
AI	Activity Index
AQP1	Aquaglyceroporin 1
BMDMs	bone marrow-derived macrophages
CC_50_	50% cytotoxic concentration
CL	cutaneous leishmaniasis
DCL	diffuse cutaneous leishmaniasis
EC_50_	50% effective concentration
MCL	mucocutaneous leishmaniasis
SbIII	trivalent antimony
SbV	pentavalent antimony
Sb	antimony
SbIII	trivalent antimony
SbV	pentavalent antimony
WHO	World Health Organization
