# Supplementary material for: Differential Susceptibility to Antimony in Strains and Clinical Isolates of Leishmania amazonensis from Brazil: In Vitro and In Vivo Studies and Implications for Drug Response and Treatment Failure
Source: Pathogens. 2026 Feb 15;15(2):220. doi: 10.3390/pathogens15020220 (PMC12943442; doi:10.3390/pathogens15020220)
Supplement: Supplementary file 1 [file pathogens-15-00220-s001.zip › pathogens-4087828-supplementary.pdf]

SUPPLEMENTARY MATERIALS

Differential Susceptibility to Antimony in Strains and Clinical Isolates of *Leishmania amazonensis* from Brazil: *In Vitro* and *In Vivo* Studies and Implications for Drug Response and Treatment Failure

Victor de Sousa Agostino ¶, Leonardo F. Geres ¶, Stéphane de la Roca, Beatriz T. de Moraes, Juliana I. Aoki, Elizabeth M. Coser, Nilson Branco, Adriano C. Coelho #

Departamento de Biologia Animal, Instituto de Biologia, Universidade Estadual de Campinas (UNICAMP), Campinas, Brazil.

¶ These authors contributed equally to this work.

# Corresponding author: Departamento de Biologia Animal, Instituto de Biologia, UNICAMP, Rua Monteiro Lobato, 255, Campinas 13083-862, Brazil. E-mail address: accoelho@unicamp.br (A. C. Coelho).

Contents

Figure S1 ______ 3
Figure S2 ______ 3
Figure S3 ______ 4
Figure S4 ______ 5

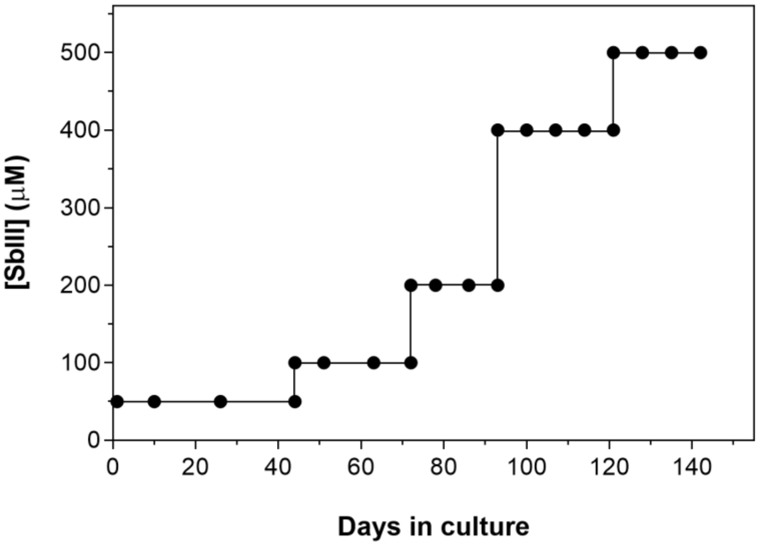

Figure S1. Schematic representation of *in vitro* selection of Sb-resistant promastigotes through stepwise selection. Each passage of the parasite population in culture is indicated by black circles.

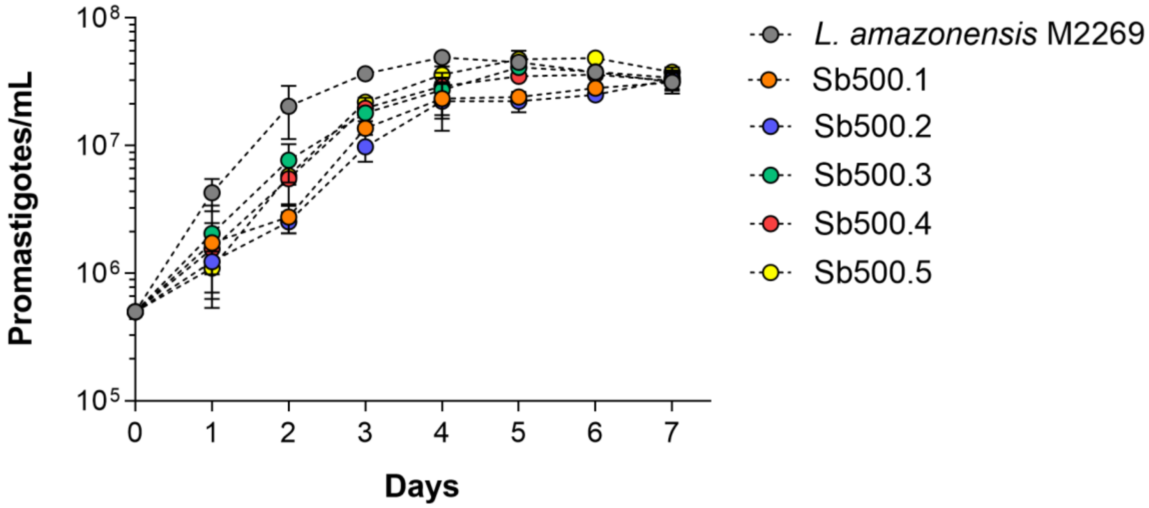

Figure S2. Promastigote proliferation of the M2269 strain, and Sb-resistant lines (Sb500.1 to Sb500.5). Promastigotes (5×10^5/mL) were cultivated at 25˚C in M199 and the number of parasites was determined daily for 7 days.

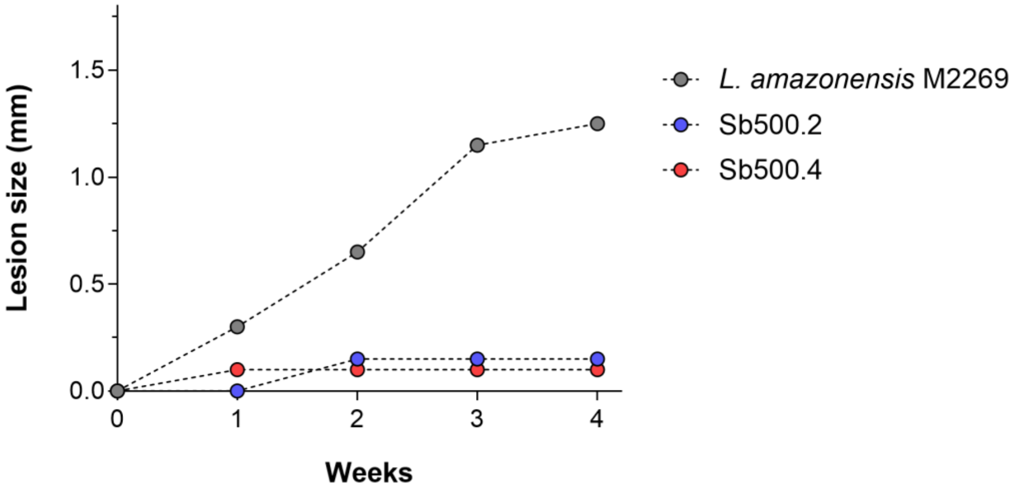

Figure S3. Progression of lesion size in infected mice with the M2269 strain and Sb-resistant clonal lines (Sb500.2 or Sb500.4) over the weeks. Lesion size was measured weekly with a caliper.

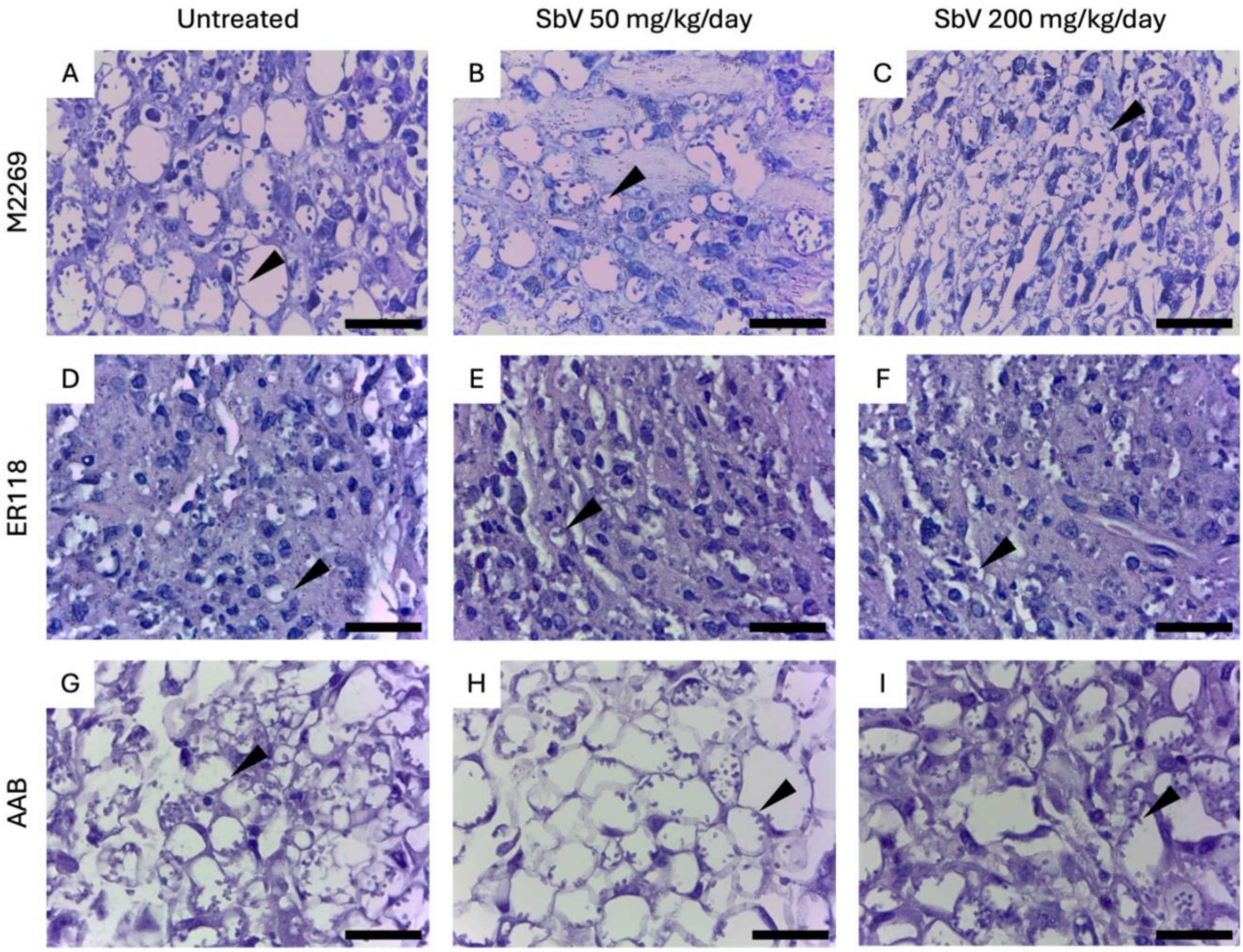

Figure S4. Histological analysis of the infected mice with *L. amazonensis* M2269 strain, or the ER118 and AAB clinical isolates. Animals were euthanized at the end of the treatment with SbV (7th week post-infection) and then infected hind footpad fragments were isolated, washed with PBS, fixed with formalin and processed with paraffin. Sections were stained with haematoxylin-eosin and then visualized in an optical microscope. Images of untreated and treated animals with 50 and 200 mg/kg/day of SbV and infected with *L. amazonensis* M2269 strain (A, B and C, respectively), ER118 (D, E and F, respectively) or AAB clinical isolate (G, H and I, respectively). Arrows heads indicate amastigotes inside macrophage vacuoles. Bar: 40 µm.
